# Influence of Toothed Rail Parameters on Impact Vibration Meshing of Mountainous Self-Propelled Electric Monorail Transporter

**DOI:** 10.3390/s20205880

**Published:** 2020-10-17

**Authors:** Yue Liu, Tiansheng Hong, Zhen Li

**Affiliations:** 1 College of Engineering, South China Agricultural University, Guangzhou 510642, China; liuyue@stu.scau.edu.cn (Y.L.); tshong@scau.edu.cn (T.H.); 2 Division of Citrus Machinery, China Agriculture Research System, Guangzhou 510642, China; 3 College of Electronic Engineering, South China Agricultural University, Guangzhou 510642, China

**Keywords:** monorail transporter, meshing impact, vibration, roller-tooth transmission, toothed rail design

## Abstract

In order to reduce the vibration of mountain self-propelled electric monorail transporters (MSEMT) caused by the impact of the meshing of roller gear with toothed rail (MRGTR), and to improve the stability and safety of monorail transporters, this paper theoretically analyzed the MRGTR mechanism of toothed monorail transporters as well as established the MSEMT displacement model and its instantaneous velocity model. The vibration signals of MSEMT with four different parameters of toothed rail were collected by the acceleration sensor and signal acquisition system. The signals were analyzed by the Hilbert envelope demodulation method to investigate the influence of toothed rail parameters on meshing impact vibration. Moreover, taking the vibration acceleration amplitude of MSEMT and the vibration attenuation time of meshing impact as evaluation indexes, a test based on the three-factor and two-level orthogonal test was engaged with factors of toothed rail pressure angle, the ratio of *L*—the chord length of two adjacent roller centers of a roller gear—and rack pitch *p* (wheel-tooth ratio) and the load mass of the MSEMT. It showed that the impact of MRGTR was the main excitation source of the vibration of MSEMT. The pressure angle and wheel-tooth ratio both have a significant impact on the smooth operation of MSEMT, the latter to a greater extent. So did the interaction between wheel-tooth ratio and load mass. The amplitude of the characteristic frequency of the MSEMT decreased with the growth of the pressure angle. When the wheel-tooth ratio was cosα, the number of the characteristic frequency was less than that when it was 1, and the amplitude became smaller too. When the pressure angle was 15, the amplitude of vibration acceleration characteristic frequency decreased as a consequence of load mass increasing. At the pressure angle of 25, the amplitude of characteristic frequency decreased with the increase of load mass if the wheel-tooth ratio was 1, and the opposite result occurs in the case when the wheel-tooth ratio was cosα. This paper provides a theoretical basis and reference for improving the impact vibration of MRGTR and optimizing the design of the toothed rail.

## 1. Introduction

The transportation network in hilly and mountainous planting areas is imperfect, and forestry transportation and agricultural materials transportation is mainly done by manpower, which is inefficient and labor-intensive [1,2]. Mechanized transportation has become a core demand for agriculture and forestry operations in hilly and mountainous areas [3]. Mountain monorail transporters have the characteristics of simple structure, flexible rail laying, easy installation, and good operating performance [4]. In recent years, experts have conducted a lot of research on mountain monorail transporters and developed a variety of mountain monorail transporters. It improved the efficiency of agriculture and forestry operations in hilly areas as well as promoted the development of agricultural and forestry mechanization in hilly areas. The research and development of a multi-purpose mountain monorail transporter [5,6] have effectively reduced the labor intensity of agricultural operations in steep slope orchards and improved the production efficiency of each production link [7,8].

Experts have not only developed multi-purpose mountain monorail transporters but also conducted extensive basic research on its key components. As far as the transmission mechanism of mountain monorail transporters is concerned, the optimal power contribution of a double-drive monorail transporter is calculated [9], the transmission structure of a double-drive monorail transporter is improved [10], the maximum torque of the driving axle is reduced, the maximum compression stress of the toothed rail is decreased, the wear of the rack is reduced, the service life is lengthened and the safety of the toothed rail is improved. The reliability of rail structure under different working conditions and environments is studied in detail, and the rationality and the safety of the rail structure are tested [11]. To investigate the effect of different tooth profiles on the mechanical properties of mountain monorail transporters, an experiment is carried out with tooth forms, rail gradients, and angular velocity as experiment factors and with the driving torque as the assessment index [12]. It shows that the sprocket toothed rack provides the best comprehensive performance.

**Figure 1:**
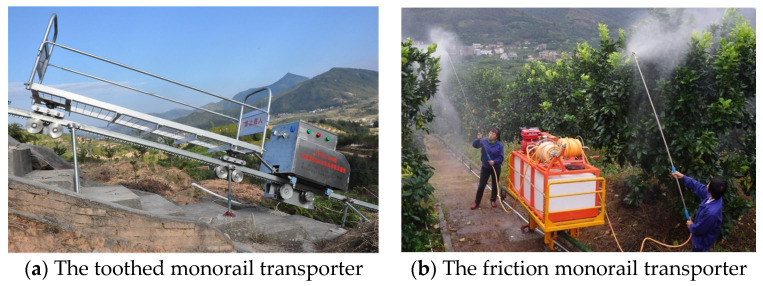
Two types of mountainous monorail transporters.

According to different transmission modes, there are two types of existing mountain self-propelled electric monorail transporters (MSEMT) i.e.,toothed monorail transporters and friction monorail transporters [13], as shown in Figure 1. Toothed monorail transporters transmit power through the meshing of roller gear with toothed rail (MRGTR), which has the characteristics of strong bearing capacity, high stability, simple structure, and simple processing, etc. It is widely used in agricultural materials transportation in mountain orchards. However, due to the influence of meshing error, machining error, and elastic deformation under load, the meshing impact occurs in the process of MRGTR [14]. The periodic vibration excitation of the MSEMT system caused by the meshing impact will easily lead to problems such as fruit quality degradation, stability degradation of MSEMT, and smoothness of whole vehicle operation. There are potential safety hazards certainly, and the necessary theoretical basis guidance is lacking in the design process. Therefore, it is of great significance to carry out MRGTR analysis to study the smoothness, mechanical properties, and safety of MSEMT. At present, there is less analysis and research on the MRGTR of MSMET than on the roller-rack and pinion system [12,15,16]. In respect of the mechanism motion, the motion principle of the roller-rack and pinion system was studied by vector analysis method, and the meshing characteristics of roller-rack and pinion systems were obtained. Then, the simulation analysis is carried out by using virtual prototype technology, and the simulation analysis is compared with the theoretical results for error analysis [17]. To avoid undercut that occurs at the dedendum, the optimized profile shifting is discussed in relation to the contact factor and pressure angle. As a result, an improved roller rack type trochoidal gear assembly is developed and tested for accuracy and smoothness of motion [18]. Then, the internal gear type trochoidal corner curve rack system is developed based on roller rack type trochoidal gear assembly, which eliminates the backlash on the connecting section of a straight rack and a corner rack by modifying the profile parameters [19]. The mechanical properties and service life were analyzed in terms of the bending strength stress of the tooth root using an involute tooth profile. And the bending strength geometric coefficient of the roller-rack and pinion system is determined, which provides a new solution for solving the bending strength problem of this system [20]. To explore the influence of load stress factors on gear noise and gear surface fatigue limits, the accurate tooth profile and the non-undercut condition satisfying the required performance is proposed with the introduction of the tooth profile shift coefficient [21,22]. The above researches provide a theoretical reference for MRGTR analysis and stability of MSEMT.

**Figure 2:**
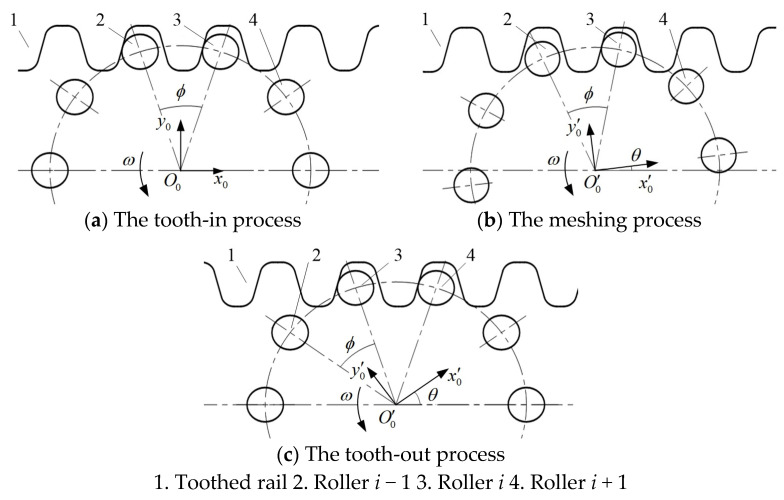
The meshing of the roller gear with a toothed rail.

The existing MSEMT often use an involute toothed rail to mesh with the roller gear to move on the toothed rail. In this process, the involute rack rail is fixed, and the movement of MSEMT along the toothed rail is caused by the implicated motion generated by MRGTR. In the power transmission process, MRGTR is divided into three processes: tooth-in, meshing, and tooth-out process depending on the mutual position relationship between the rollers’ curved surface and the tooth profile inclined surface of the involute rack, as shown in Figure 2. Without considering the influence of the left and right swing of MSEMT, there is a line of contact between the roller and the tooth surface of the toothed rail. The meshing contact line is parallel to the central axis of the roller, so the process of MSEMT can be simplified as a plane meshing problem [23] for analysis.

This paper took MSEMT as the research object, analyzed its MRGTR mechanism, and established the model of displacement and instantaneous velocity model of MSEMT. The vibration response characteristics of MSEMT with meshing impact excitation were analyzed as well. Then, the meshing vibration test was conducted, which takes the load mass and toothed parameters as the investigation factors, and the vibration acceleration signal in the displacement direction of MSEMT, and the vibration attenuation time of the meshing impact as the evaluation indexes. Furthermore, the influence of toothed rail parameters on the meshing impact vibration of MSEMT was studied.

## 2. MRGTR Mechanism and Meshing Impact Vibration Analysis

### 2.1. MRGTR Mechanism Analysis

#### 2.1.1. Displacement Model of MSEMT

**Figure 3:**
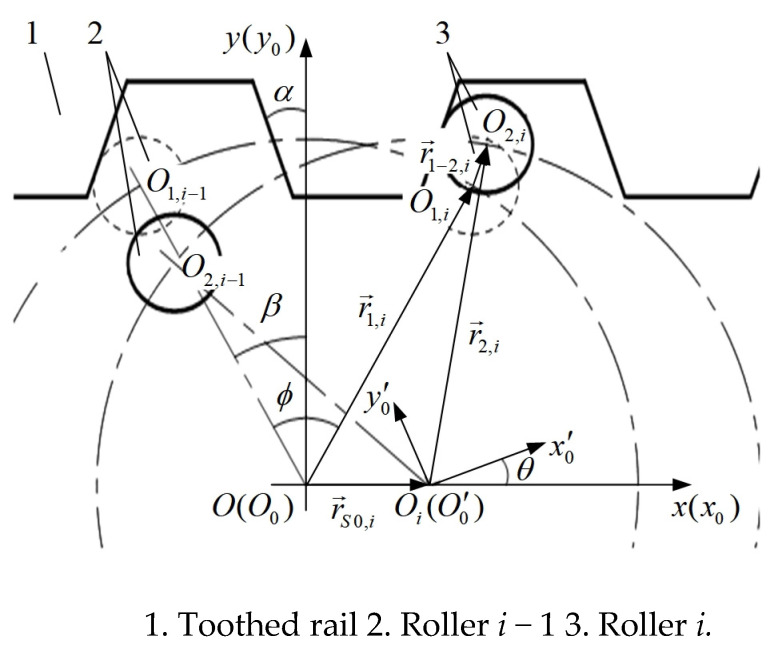
Position of the roller gear after rotation *θ.*

Assume that the initial position is where the roller *i* comes into contact with the toothed rail (i.e., the tooth-in process), as shown in Figure 2a. The fixed coordinate *xoy* takes the roller gear center at the initial position as the coordinate center *O*, the rail extension direction as the *x*-axis, and the straight line perpendicular to the *x*-axis in the plane as the *y*-axis. The roller gear rotates counterclockwise around the coordinate center *O* at a given angular speed *ω*, as shown in Figure 3. After the roller gear rotation *θ*, it is only the roller *i* that contacts with the toothed rail. The MRGTR produces an implicated motion so that the roller gear center *O* moves to *O_i_* with the vector ${\vec{r}}_{S0,i}$, which is a displacement distance of the MSEMT. The initial position of the center of the roller *i* is *O*_1,*i*_, which is represented by vector ${\vec{r}}_{1,i}$. The center of roller *i* is *O*_2,*i*_, which is represented by vector ${\vec{r}}_{2,i}$. The center of roller *i* moves from *O*_1,*i*_ to *O*_2,*i*_ is represented by vector ${\vec{r}}_{1-2,i}$. According to the Euler formula:

(1) $$\left\{ \begin{array}{l} {\vec{r}}_{1,i}=R\cdot e^{j(\frac{\pi }{2}-\phi +\beta )}\\ {\vec{r}}_{2,i}=R\cdot e^{j(\frac{\pi }{2}-\phi +\beta +\theta )}\\ {\vec{r}}_{S0,i}=S_{0,i}\cdot e^{0}\\ {\vec{r}}_{1-2,i}=aa_{1}\cdot e^{j(\frac{\pi }{2}-\alpha )} \end{array}\right.$$

where *R* is the central circle radius of the rollers inside the roller gear, m; ϕ is the circumferential angle corresponding to the connecting line between the centers of two adjacent rollers, °; *β* is the meshing angle at the initial position, °; *S*_0,1_ is the distance of the MSEMT moving along the toothed rail, m; *aa*_1_ is the distance of roller *i* moving along the rack tooth surface, m; *α* is the pressure angle of toothed rail, °.

According to the vector superposition relation:

(2) $${\vec{r}}_{S0,i}+{\vec{r}}_{2,i}={\vec{r}}_{1-2,i}+{\vec{r}}_{1,i}$$

As roller *i* moves along the rack tooth surface, the direction angle of vector ${\vec{r}}_{1-2,i}$ and the pressure angle *α* of the rack are mutually complementary, then:

(3) $$\cot\alpha =\frac{x_{1-2,i}}{y_{1-2,i}}$$

Combining Equations (1)–(3), the *S*_0,*i*_ is calculated as:

(4) $$S_{0,i}=\frac{R}{\cos\alpha }\cdot \left[\sin\left(\theta +\alpha +\beta -\phi \right)-\sin\left(\alpha +\beta -\phi \right)\right]$$

When roller *i* is engaged with the toothed rail for critical separation, roller *i* + 1 is engaged with the toothed rail for critical contact, as shown in Figure 2c. The rotation angle of the roller gear is ϕ, and the *S*_0,*i*_ is R[sin(α+β)−sin(α+β−ϕ)]/cosα. The roller gear state is consistent with the initial state, which means that when the roller gear rotation angle is an integral multiple of ϕ, its state is consistent with the initial state. Therefore, the displacement equation of the MSEMT moving from *O*_*i*−1_ to *O_i_* during the meshing between roller *i* and the toothed rail is as follows:

(5) $$S_{i-1,i}=\frac{R}{\cos\alpha }\cdot [\sin(\theta +\alpha +\beta -i\phi )-\sin(\alpha +\beta -\phi )]$$

Consequently, the displacement of the roller gear from the initial position *O* to *O_i_* in a rotation cycle can be calculated by accumulating the displacement distances of the MSEMT during the meshing of each roller, namely:

(6) $$\begin{array}{cc} S_{0,n} & =\sum_{1}^{i}S_{i-1,i}\\ & =\frac{R}{\cos\alpha }\cdot [\sin(\theta +\alpha +\beta -i\phi )-i\cdot \sin(\alpha +\beta -\phi )+(i-1)\cdot \sin(\alpha +\beta )] \end{array}$$

As the rotation cycle of the roller gear increases, the displacement equation of the MSEMT along the rail is:

(7) $$\begin{array}{cc} S & =\sum_{1}^{i}S_{i-1,i}+(N-1)\cdot S_{0,n}\\ & =\frac{R}{\cos\alpha }\{\sin[\theta +\alpha +\beta -(zN-z+i)\phi ]-(zN-z+i)\sin[\alpha +\beta -\phi ]+(zN-z-N+i)\sin(\alpha +\beta )\} \end{array}$$

where *N* is the number of cycles the roller gear has rotated and *z* is the number of rollers inside the roller gear.

#### 2.1.2. The Meshing Angle β at Initial Position

**Figure 4:**
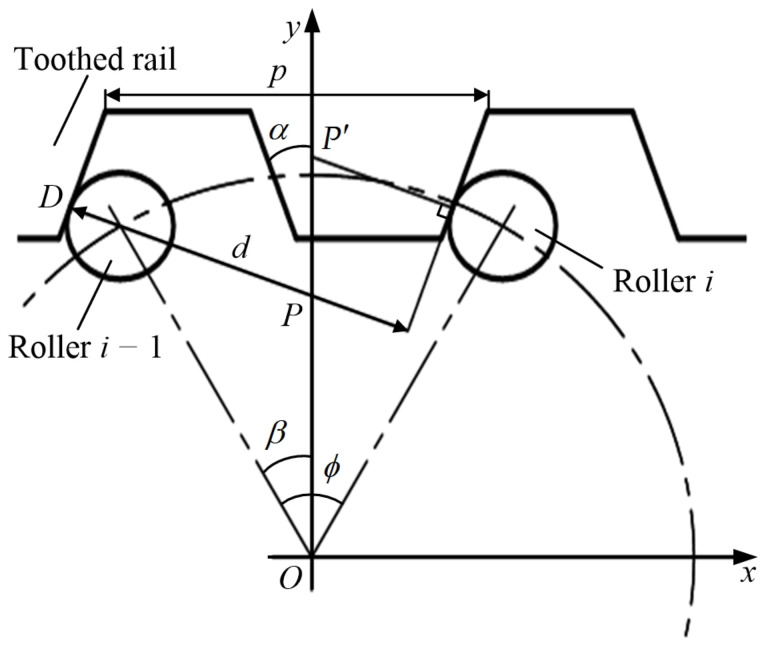
Initial position of the roller gear.

In the initial position of the roller gear, the roller *i* is in critical contact with the tooth surface, but the roller *i* − 1 is in critical separation from the tooth surface. The meshing angle *β* at the initial position is between the line of *O*_1,*i*−1_ and the roller gear center *O* and the *y*-axis, as shown in Figure 4. The contact point between roller *i* − 1 and the tooth surface is *D*. The distance between *D* and the contact tooth surface of roller *i* is *d*, then:

(8) $$d=2R\cdot \sin\frac{\phi }{2}\cdot \cos(\alpha +\beta -\frac{\phi }{2})=p\cdot \cos\alpha$$

where *p* is the pitch of rack, mm.

As Equation (8) solved, the meshing angle *β* is a positive value and β≤ϕ can be obtained as follows:

(9) $$\left\{ \begin{array}{l} \beta \;=\;\frac{\phi }{2}\;-\;\alpha \;\pm \;\arccos(\frac{1}{A}\cdot \cos\alpha )\\ A\;=\;\frac{2R\cdot \sin(\phi /2)}{p} \end{array}\right.$$

where *A* is the ratio of *L*, the chord length of two adjacent roller centers of a roller gear, and rack pitch *p* (wheel-tooth ratio).

#### 2.1.3. Instantaneous Velocity Model of MSEMT

The roller gear rotates at angular velocity ω whose rotation angle is *θ*(*t*) in t time. At this point, the displacement equation of MSEMT is shown in Equation (7). Then, the instantaneous velocity *v*(*θ*) of MSEMT can be obtained by differentiating time *t*, namely:

(10) $$\left\{ \begin{array}{c} \theta (t)\;=\;\omega \cdot t\\ v(\theta )=\frac{dS}{dt}=\frac{dS}{d\theta }\cdot \frac{d\theta }{dt}\\ \;=\frac{R\omega }{\cos\alpha }\cdot \cos[\theta +\alpha +\beta -(zN-z+i)\phi ] \end{array}\right.$$

When the rotation angle of the roller gear is an integral multiple of ϕ, *P* is the pitch point caused by a meshing of roller *i* − 1 and the tooth surface. Similarly, *P*′ is the pitch point which is resulted from a meshing of roller *i* and the tooth surface, as shown in Figure 4. If these two pitch points do not coincide, the velocity of the MSEMT will jump. On the contrary, the velocity will not jump if they do coincide.

When the rotation angle of the roller gear is $i\phi^{-}$ in the *N*-th rotation cycle, the roller *i* − 1 is in contact with the tooth surface, and the instantaneous velocity of the MSEMT is:

(11) $$v(2N\pi -2\pi \;+\;i\phi^{-})\;=\;\frac{R\omega }{\cos\alpha }\cdot \cos(\alpha \;+\;\beta )$$

However, when it is $i\phi^{+}$, the roller *i* is in contact with the tooth surface and the instantaneous velocity of MSEMT is:

(12) $$v(2N\pi -2\pi \;+\;i\phi^{+})=\frac{R\omega }{\cos\alpha }\cdot \cos(\alpha +\beta -\phi )$$

And, the variation of instantaneous velocity Δ*v* is:

(13) $$\begin{array}{c} \Delta v=v(2N\pi -2\pi +i\phi^{+})-v(2N\pi -2\pi +i\phi^{-})\\ \;=\;\frac{2R\omega }{\cos\alpha }\cdot \sin(\alpha +\;\beta -\;\frac{\phi }{2})\cdot \sin\frac{\phi }{2} \end{array}$$

Generally speaking, the rollers *z* of the roller gear is more than 4 so that sinϕ/2≠0. Therefore, if the instantaneous velocity of the MSEMT does not jump at this moment, then:

(14) $$\alpha +\beta -\frac{\phi }{2}=m\pi \;(m=\;1,\;2,\;3\ldots ),$$

Combining Equation (9) with Equation (15), if Δ*v* is 0, it can be obtained that:

(15) A=cosα,

Combining Equation (9) with Equations (13) and (14), when *A* > cos*α* as well as two adjacent rollers alternating, the instantaneous velocity jump of the MSEMT becomes smaller with the decrease of wheel-tooth ratio.

### 2.2. MRGTR Impact Vibration

The roller gear and rack mechanism is a set of devices that continuously transmits motion and power through MRGTR. However, there are manufacturing errors and installation errors of the toothed rail and roller all the time. Moreover, elastic deformation can occur during power transmission. From the kinematic analysis in the previous section, it can be seen that when Equation (15) is not satisfied, there is a leap in the instantaneous velocity of the MSEMT in cases of the tooth-in or tooth-out processes. This causes a meshing impact, which creates periodic oscillation excitation to the whole system.

#### 2.2.1. Force Analysis of the Driving Mechanism

**Figure 5:**
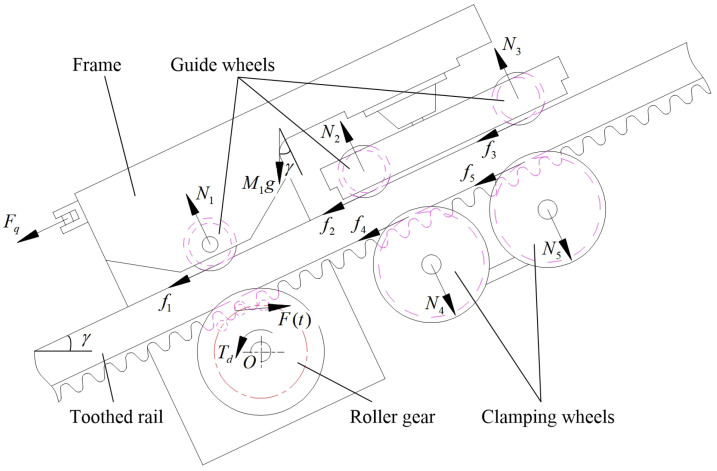
Force analysis of components of the mountain self-propelled electric monorail transporter (MSEMT).

The MSEMT is taken as the research object. The overall mass of the MSEMT without the cargo car is *M*_1_. The number of guide wheels is 3, and the number of clamping wheels is 2. When the MSEMT works, MRGTR produces a resistance torque *T_e_*, the guide wheel and clamping wheel generate friction force *f_i_* (*i* = 1, 2, 3, 4, 5) with the toothed rail, and the torque provided by the roller gear is *T_d_*. The force analysis of components is shown in Figure 5.

The moment of various forces acting on the MSEMT about point *O* can be calculated. The driving torque *T_d_* can be calculated as:

(16) $$T_{d}=T_{e}+M_{1}gl_{W}\cdot \sin\gamma +\sum_{1}^{5}N_{i}l_{i}-M_{1}gh_{W}\cdot \cos\gamma -\sum_{1}^{5}f_{i}h_{i}-F_{q}h_{q}$$

where *T_d_* is the driving torque provided by the MSEMT in operation, N∙m; *T_e_* is the resistance torque of MRGTR, N∙m; *M*_1_ is the overall mass of the MSEMT without the cargo car, kg; *g* is the acceleration of gravity, m/s^2^; *l_W_* is the horizontal distance from the center of mass of the MSEMT to the center of the roller gear, m; *γ* is the slope angle, °; *h_W_* is the vertical distance from the center of mass of the MSEMT to the center of the roller gear, m; *N_i_* is the pressure on the guide wheels and clamping wheels, N; *l_i_* is the horizontal distance from the centers of each guide wheel and each clamping wheel to the center of the roller gear, which is *l*_1_, *l*_2_, *l*_3_, *l*_4_, *l*_5_, m; *f_i_* is the friction force at the guide wheel and clamping wheel, which is *f*_1_, *f*_2_, *f*_3_, *f*_4_, *f*_5_, N; *h_i_* is the vertical distance from the center of each guide wheel and each clamping wheel to the center of the roller gear, which is *h*_1_, *h*_2_, *h*_3_, *h*_4_, *h*_5_, m; *F_q_* is the resistance produced by the loaded trailer when the MSEMT is working, N; *h_q_* is the vertical distance from the resistance caused by the loaded trailer to the center of the roller gear.

The resistance torque of MRGTR is caused by meshing force *F*(*t*) regardless of Coulomb friction. According to Hertz’s collision theorem [24], it can be known that the meshing force *F*(*t*) is always perpendicular to the meshing surface of the toothed rail, from which:

(17) $$\left\{ \begin{array}{l} T_{e}=F(t)l_{F}\cdot \cos\alpha \\ l_{F}=\sqrt{R^{2}+r^{2}-2Rr\cdot \cos\alpha } \end{array}\right.$$

where *l_F_* is the distance from the contact point of roller and rack to the center of the roller gear, m; *R* is the central circle radius of the rollers inside the roller gear, m; *r* is the radius of the roller, m.

Suppose the MSEMT has been operating for a time *t*, that roller *i* engages with the toothed rail at that point. According to the conservation of momentum, an analysis of the MSEMT, as a whole, yields:

(18) $$M_{1}v(\theta )-M_{1}v_{0}=\int_{t0}^{t}\left[F(t)\cdot \cos\alpha -F_{q}-\sum_{1}^{5}f_{i}\right]dt$$

where *v*_0_ is the velocity of the MSEMT when the roller *i* − 1 is engaged with critical separation, m/s; *t*0 is the time to reach the critical separation, s.

#### 2.2.2. Dynamic Model of Meshing Impact Vibration of MSEMT

**Figure 6:**
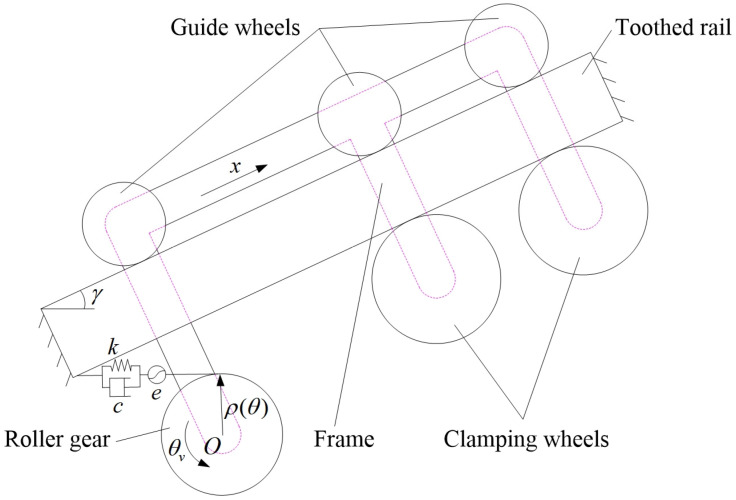
Dynamic model of the MSEMT.

To simplify the analysis, MRGTR transmission system is treated as a torsional vibration model [25] without considering the influence of elastic deformation of the roller gear transmission shaft, support bearing, guide wheel, compression wheel, and support shaft on the vibration analysis of the MSEMT. Only the influence of meshing impact on the torsion angle of the roller gear and the elastic deformation of the toothed rail caused by the meshing force are considered in this analysis. Based on the structure and working characteristics of the roller gear and rack mechanism, it is assumed that in the ideal case, *θ_v_ is* the instantaneous rotation angle, *k* is the comprehensive meshing stiffness between roller gear and toothed rail, *c* is the meshing damping coefficient, *e* is the meshing error, *ρ*(*θ*) is the curvature radius of the base circle on the roller gear, and *J* is the rotational inertia of the roller gear to the center *O*. The dynamic model of the MRGTR transmission mechanism is shown in Figure 6.

If the generalized coordinates are *q* = {*θ_v_*,*x*}. The corresponding generalized forces are $Q=\{T_{d},-\sum_{1}^{5}f_{i}-F_{q}\}$. The total kinetic energy *E_k_* of the system is composed of the kinetic energy *E_k_*_0_ of the roller gear and the kinetic energy *E_k_*_1_ of the MSEMT. The total potential energy *E_p_* of the system is a sum of the gravitational potential energy *E_p_*_0_ and the comprehensive elastic potential energy *E_p_*_1_ of the meshing. Damping potential energy is composed of work *W*_0_ from the meshing, work *W*_1_ from the resistance force *F_q_*, and work *W*_2_ from friction force *f_i_*, then:

(19) $$\left\{ \begin{array}{l} E_{k}=\sum E_{ki}=\frac{1}{2}(J{\dot{\theta }}_{v}{}^{2}+m_{1}{\dot{x}}^{{}^{2}}+m_{2}{\dot{x}}^{2})\\ E_{p}=\sum E_{pi}=\frac{k}{2}{\left[\theta_{v}\rho (\theta )-e-x\right]}^{2}+M_{1}gx\cdot \sin\gamma \\ W=\sum W_{i}=c[\dot{e}+\dot{x}-{\dot{\theta }}_{v}\rho (\theta )]\cdot (e+x-\theta_{v}\rho (\theta ))-F_{q}x-\sum_{1}^{5}f_{i}x \end{array}\right.$$

where *m*_1_ is the mass of the roller gear, kg; *m*_2_ is the mass of the MSEMT with roller gear removed, and its value is *M*_1_ − *m*_1_, kg.

According to the theory of gearing, the curvature radius *ρ*(*θ*) of the base circle on the roller gear is the curvature radius *ρ*_0_(*θ*) of the instantaneous centerline of the roller inside the roller gear, then:

(20) $$\rho (\theta )=\rho_{0}(\theta )=\frac{dS}{d\theta }=\frac{R}{\cos\alpha }\cdot \cos(\theta +\alpha +\beta -\phi )$$

For the mechanical system, the dynamic of the system can be modeled by using Lagrange’s equations of motion for a generally complete constraint system [26], namely:

(21) $$\frac{d}{dt}\left(\frac{\partial E_{k}}{\partial {\dot{q}}_{i}}\right)-\frac{\partial E_{k}}{\partial q_{i}}+\frac{\partial W}{\partial q_{i}}+\frac{\partial E_{p}}{\partial q_{i}}=Q_{i}$$

where *q_i_* is a generalized coordinate system and *Q_i_* is a generalized force.

The dynamic equation of the MSEMT can be obtained by solving the Equations (16)–(21), that is, satisfying:

(22) $$M\ddot{q}+C\dot{q}+Kq=Q$$

where *M* is the mass matrix, *C* is the damping matrix, *K* is the stiffness matrix, and vector *Q* is the generalized force. 

The calculation results of Equation (22) are as follows:

$$M=\left[\begin{array}{cc} J & 0\\ 0 & m_{1}+m_{2} \end{array}\right],\;C=\left[\begin{array}{cc} c\rho {\left(\theta \right)}^{2} & -c\rho (\theta )\\ -c\rho (\theta ) & c \end{array}\right],\;K=\left[\begin{array}{cc} k\rho {\left(\theta \right)}^{2} & -k\rho (\theta )\\ -k\rho (\theta ) & k \end{array}\right]\;\mathrm{and}\;Q=\left[\begin{array}{c} T_{d}+ke\rho (\theta )+c\dot{e}\rho (\theta )\\ -ke-c\dot{e}-M_{1}g\sin\gamma \end{array}\right].$$

Equation (22) is a second-order linear differential equation system. According to the differential equation theory, the vibration model equation of the MSEMT can be obtained by determining the constraint conditions.

#### 2.2.3. Numerical Validation

**Figure 7:**
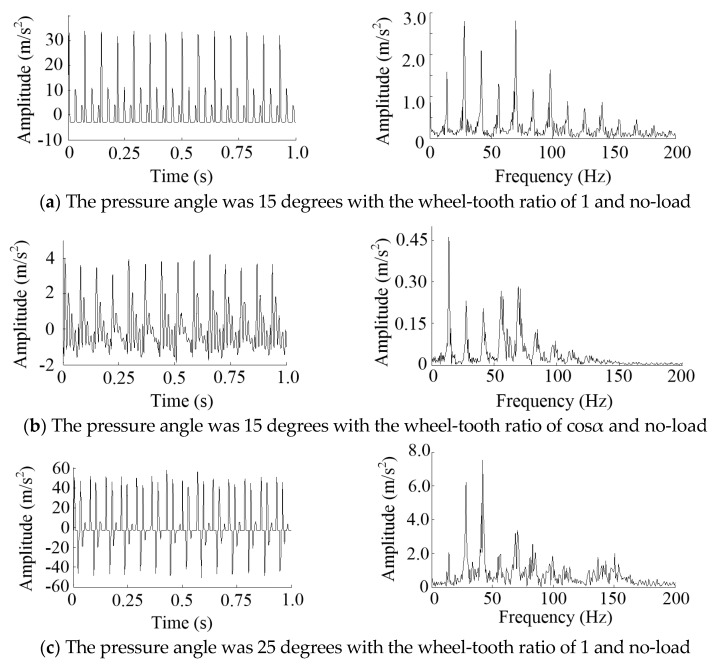

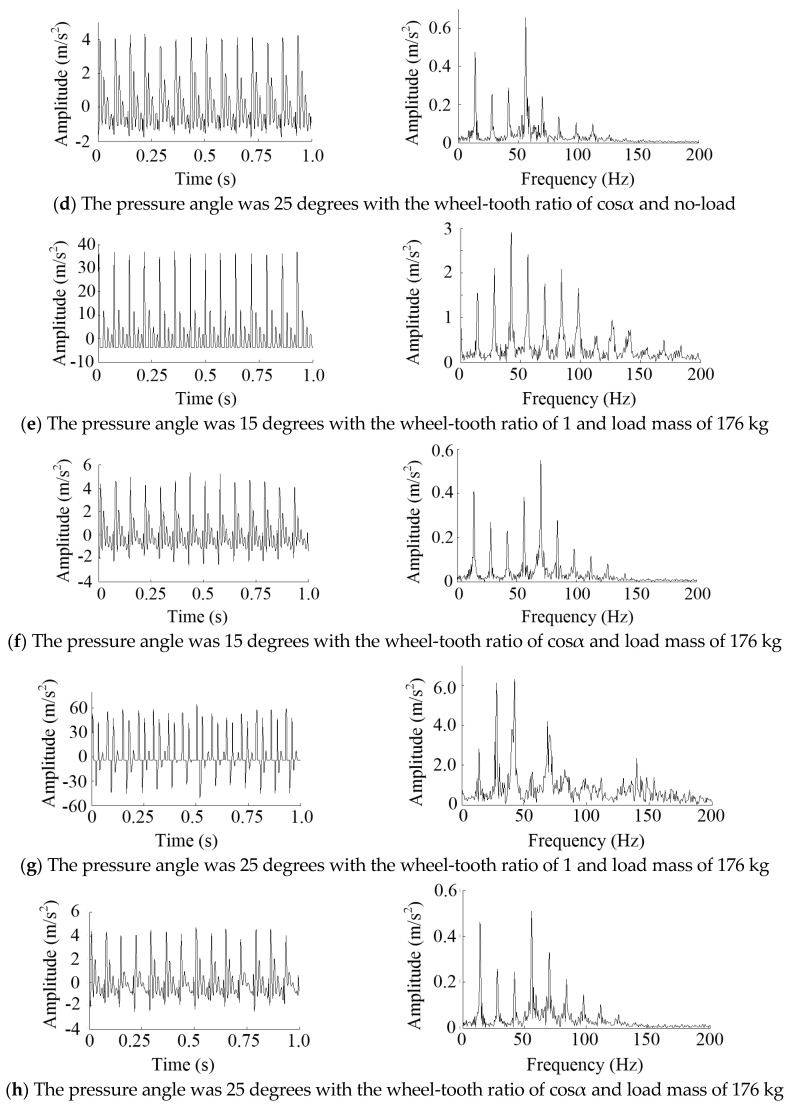
Time-domain and spectral of simulation results.

A 1000 point long numerical signal, simulating the real behavior of the MSEMT, was generated in order to provide a validation of the excitation source causing the vibration of the MSEMT. The overall mass of the MSEMT without the cargo car was set to 108 kg. The moment of inertia *J* was 15.25 $\mathrm{kg}\cdot m^{2}$. The roller gear and rack mechanism were similar to the rack and pinion mechanism. According to the reference [27], the mean meshing stiffness can be calculated. The damping ratios for meshing were generally 0.03 to 0.17, according to R. Kasuba and K.L. Wang [27]. The meshing damping was calculated through the mean meshing stiffness and the damping ratios [28]. Hypothetically speaking, the meshing error was 0 m at the ideal condition. The time-varying curvature radius *ρ*(*θ*) can be obtained by Formula (20). The rotational speed of the roller gear was 1.4 r/s. The number of rollers was 10. The meshing frequency of 14 Hz was easily obtained. The result of the simulation is shown in Figure 7.

The results of the frequency analysis showed that the vibration peaks appeared at 14 Hz and its octave. It means that the excitation source was the impact of meshing. The pressure angle of 15 degrees had a smaller amplitude of characteristic frequency. So did the wheel-tooth ratio of cosα. There was a small variation in the amplitude of characteristic frequency with the different load mass. With the increase of load mass, the amplitude of characteristic frequency decreased.

## 3. Materials and Methods

### 3.1. Test Material

**Figure 8:**
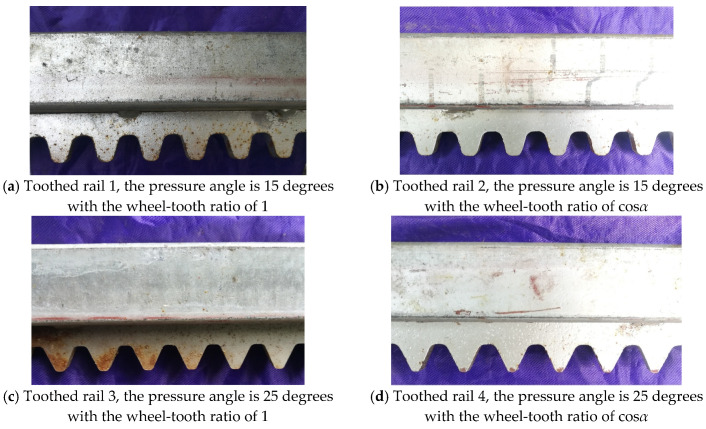
Toothed rails for the testing.

To explore the influence of the involute toothed rail pressure angle, wheel-tooth ratio, and load mass on the meshing impact vibration of the MSEMT, the meshing vibration test of the MSEMT was carried out in a laboratory where 45 steel was used as the material for the rack. According to the design requirement of the Mechanical Design Manual [29], and referring to the original rack parameters of 7SYZDD-200 MSEMT, 4 involute racks with different parameters were designed and machined by laser cutting technology. Then, these racks were spot welded on a galvanized square pipe with a cross-section of 50 mm × 50 mm × 3 mm (length × width × thickness) to make the toothed rails for the test, as shown in Figure 8. The length of a single toothed rail is about 6 m. These racks of toothed rails are a non-standard design. Their pitches are calculated from the modulus. The rack parameters of each toothed rail are shown in Table 1. Six buckets with a volume of 260 mm × 260 mm × 480 mm (length × width × height) are used as the load mass. The total mass of these 6 buckets with water is about 176 kg, which shall be weighed and recorded before each test.

### 3.2. Test Platform and Equipment

**Figure 9:**
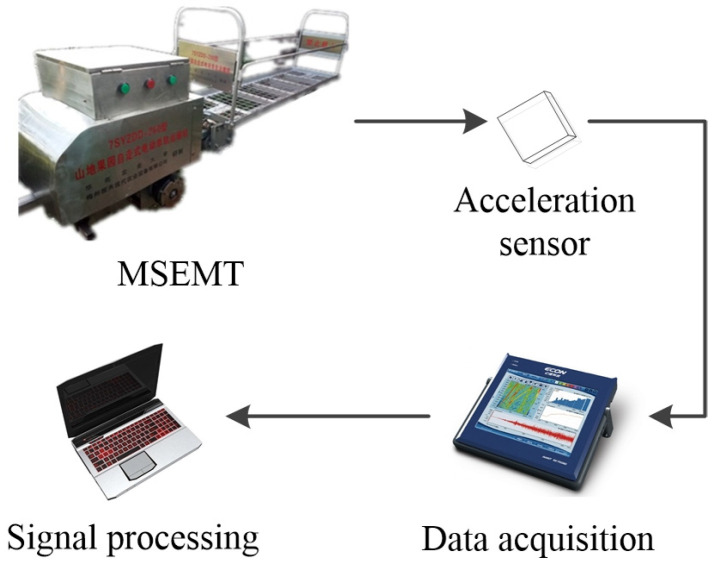
Test system.

**Figure 10:**
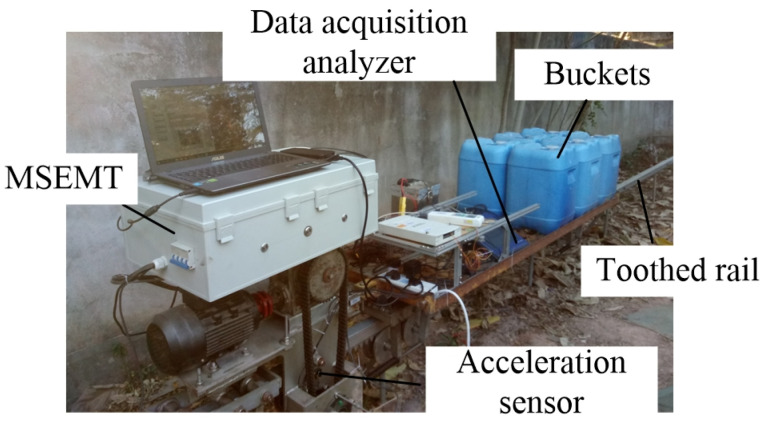
The position of the acceleration sensor.

The experiment was conducted in the laboratory, which was located at the Division of Citrus Machinery of the China Agriculture Research System of South China Agricultural University. The test platform was mainly composed of testing the MSEMT, the toothed rail, and the measurement system. The MSEMT testing adopted a 7SYZDD-200 monorail transporter developed by the Division of Citrus Machinery of the China Agriculture Research System of South China Agricultural University. A lithium battery was its power energy with a rated load of 200 kg. Its specific parameters are shown in Table 2. The testing toothed rail was about 18 m long, which was composed of three toothed rails each about 6 m long. The MSEMT started slowly at first with a start-up time of about 3 s measured by the preliminary test. To ensure the acquisition of meshing impact vibration data of the MSEMT in stable operation, an area of 6 m long was taken as the test preparation area from each end of the toothed rail, with a remaining area of 6 m long for data acquisition. The measurement system included a portable data acquisition analyzer (ECON, AVANT MI-7008D) and a triaxial acceleration sensor (EA-YD-152). The connection mode is shown in Figure 9. The portable data acquisition analyzer has 8 voltage /IEPE input channels, which can support up to a sampling rate of 102.4 kHz. In this paper, a triaxial acceleration sensor was installed above the driving axle of the MSEMT with its *x*-axis corresponding to the forward direction of the MSEMT, as shown in Figure 10. Moreover, the *x*-axis direction vibrations were measured and analyzed. In order to collect the meshing impact vibration signal, the sampling rate was set at 3200 Hz and, therefore, constituted the vibration detection unit of the MSEMT.

### 3.3. Experiment Design

**Figure 11:**
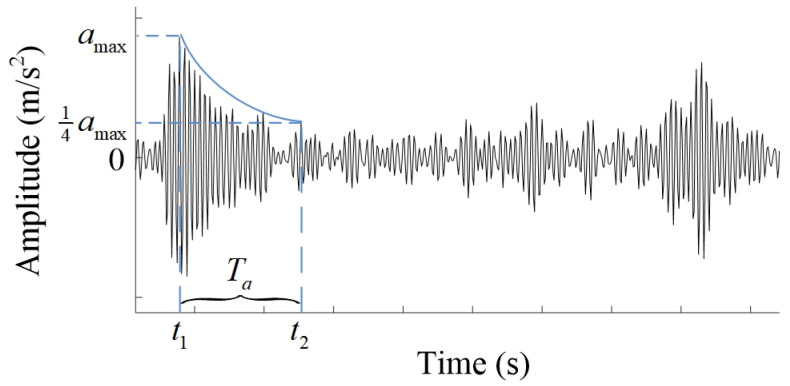
Definition of vibration attenuation time.

Based on the above theoretical analysis, without the roller gear structure changed, the vibration of the MSEMT caused by meshing impact was related to the toothed rail pressure angle α, the wheel-tooth ratio, and the load mass *M*_2_. Moreover, they have an interactive influence. Therefore, the vibration characteristics of the MSEMT were analyzed by a whole factor experiment with the average maximum amplitude of vibration acceleration and vibration attenuation time of meshing impact as evaluation indexes. In this paper, the vibration attenuation time was defined as the time consumed from the start of impact collision to the time when the peak acceleration amplitude drops to 1/4 of the maximum acceleration amplitude in the impact process, as shown in Figure 11. Considering the interaction among various factors, an L_8_(2^7^) orthogonal test table was used in the test. Each level of any factor (A, B, C) was used 4 times in this test, which means its chance of occurrence was evenly balanced. The combinations of levels of any two columns were (1, 1), (2, 2), (1, 2), (2, 1). They all had a chance of occurring twice [30]. The three factors and two levels tested are listed in Table 3.

### 3.4. Evaluation and Calculation Method

In order to accurately measure the vibration characteristics of the MSEMT caused by meshing impact, the test of the toothed rail with each parameter was repeated 3 times under different load masses, respectively. Each time the start position of the MSEMT displaced 1 m forward relative to the previous one. The data within 4 s after the smooth operation of the MSEMT was fetched for test data analyzing. To obtain the signal accurately and intuitively, the vibration signal of the MSEMT was collected with filtering. The filter band was selected by the ratio of the cyclic content (RCC) method. The RCC has proven not only more robust than the kurtosis for the selection of the optimal spectral band for demodulation, but also a well-performing prognostic tool [31]. An estimate of RCC is from the reference [31], it can be calculated as follows:

(23) $$RCC_{l,h}^{p,q}=\frac{\frac{1}{N}\sum_{n=1}^{N}{\left\{({\left|x[n]⊗FILTER(l,h)\right|}^{2})⊗FILTER(p,q)\right\}}^{2}}{\frac{1}{N}\sum_{n=1}^{N}{\left({\left|x[n]⊗FILTER(l,h)\right|}^{2}\right)}^{2}}$$

where (*l*,*h*) are the lower and upper bounds of the 4-step Butterworth’s filter applied to calculate the envelope signal; (*p*,*q*) are the bounds of the cyclic passband for the integration of the kurtosis contribution. 

Because the theoretical revolution speed of the roller gear is between 81.67 rpm and 93.33 rpm with 10 rollers, the frequency of MRGTR was calculated between 13.61 Hz and 15.55 Hz. To calculate the RCC, the narrowband range was set to between 13 Hz and 16 Hz, which was used for the cyclic passband. The filter passbands were a constant bandwidth 0.15 times the Nyquist frequency and with an overlap between two subsequent bands of 2/3 the bandwidth. 

The processed vibration signals were segmented and analyzed. The maximum acceleration amplitude *a_i,max_*, the meshing period *T_i_*_,_ and the attenuation time of the vibration *T_i,a_* during each meshing period were marked and recorded. Assuming that the MSEMT operates smoothly for 4 s, the average maximum acceleration amplitude $\bar{a}$, the average meshing period $\bar{T}$, and the average attenuation time of vibration ${\bar{T}}_{a}$ in this 4 s can be calculated as follows:

(24) $$\left\{ \begin{array}{l} \bar{a}=\frac{1}{3}\sum_{1}^{3}\left(\frac{1}{N_{S}}\sum_{1}^{N_{S}}a_{i,\max}\right)\\ \bar{T}=\frac{1}{3}\sum_{1}^{3}\left(\frac{1}{N_{S}}\sum_{1}^{N_{S}}T_{i,\max}\right)\\ {\bar{T}}_{a}=\frac{1}{3}\sum_{1}^{3}\left(\frac{1}{N_{S}}\sum_{1}^{N_{S}}T_{i,a}\right) \end{array}\right.,$$

where *N_S_* is the number of meshing cycles in 4 s of operation.

The average maximum acceleration amplitude and average attenuation time of vibration for each test were recorded and analyzed using SPSS software 30.

## 4. Results and Analysis

### 4.1. Processing and Analysis of Vibration Signal Results

**Figure 12:**
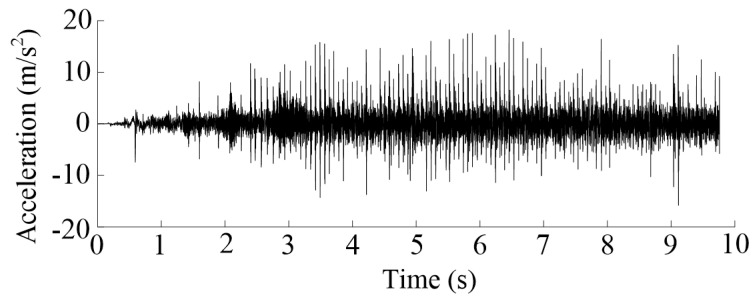
Time-domain diagram of the vibration acceleration signal. Note: The time-domain diagram was collected with the testing of toothed rail 1 and a 0 kg load mass.

The time-domain diagram of the vibration acceleration signal is shown in Figure 12. In the first 3 s, the amplitude of peak acceleration increased gradually. However, the interval time between adjacent peaks gradually decreased. This is an acceleration process for the MSEMT, which is almost consistent with the results obtained from the pre-test. After 3 s operation, the interval time between adjacent peaks tended to be stable, but the value of peak changed obviously with time, and there was no obvious rule.

**Figure 13:**
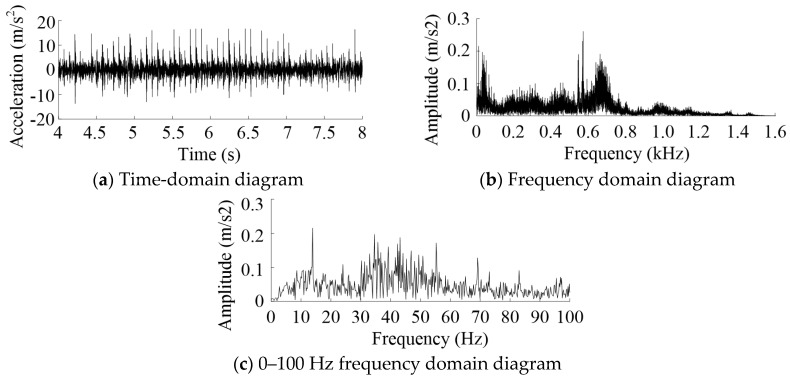
4~8 s Vibration acceleration signal analysis. Note: The acceleration signal was acquired with the testing of toothed rail 1 rail and load mass of 0 kg.

The vibration acceleration signal of 4~8 s was processed and analyzed. During this time, the interval time between adjacent peaks did not change much, as shown in Figure 13a. The vibration acceleration signal was acquired with the toothed rail 1 as the test rail and load mass of 0 kg. It was analyzed by frequency analysis. The spectrum diagram is shown in Figure 13b. The spectrum of the low-frequency range is shown in Figure 13c. The low-frequency vibrations were mainly concentrated around 13.87 Hz and 55.37 Hz, and there was a frequency doubling relationship between them. The theoretical frequency of MRGTR was between 13.61 Hz and 15.55 Hz. The high-frequency vibration was mainly concentrated around 572.9 Hz and 662.6 Hz and did not show an octave relationship with the frequency of MRGTR. However, the above results did not make it clear which excitation source is causing the vibration of the MSEMT. In order to pinpoint it, the Hilbert envelope demodulation was performed on the filtered vibration acceleration signal [32].

**Figure 14:**
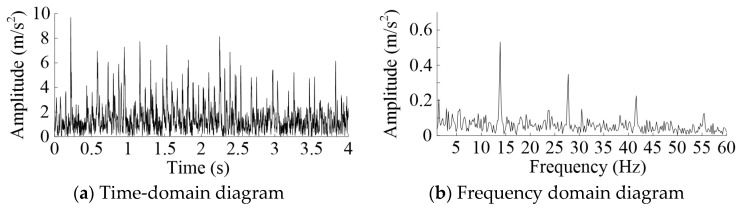
Hilbert envelop demodulation analysis of the filtered vibration acceleration signals.

The filtered vibration acceleration signals were analyzed by Hilbert envelope demodulation and frequency analysis, as shown in Figure 14. The results of the frequency analysis showed that the vibration peaks appeared at 13.87 Hz, 27.73 Hz, 41.6 Hz, and 55.47 Hz. The peak characteristics of the filtered vibration signal were more prominent than the original one. All frequencies had a frequency doubling relationship with the frequency of MRGRT. Moreover, the amplitude decreased with the increase of frequency doubling. The toothed rails for other experimental conditions showed such a change rule too.

The vibration signals of all test groups were filtered and analyzed by Hilbert envelope demodulation analysis. The peak frequency and amplitude are shown in Table 4. With the increase of frequency doubling of vibration frequency, the amplitude of the vibration acceleration signal decreased gradually, and the decreasing rate slowed down. When the pressure angle was 15 degrees, the amplitude corresponding to each peak frequency decreased with the increase of the load mass. At a pressure angle of 25 degrees, the conclusions varied depending on the wheel-tooth ratio. When the wheel-tooth ratio was cosα, the amplitude of each peak frequency increased with the increase of the load mass. When the wheel-tooth ratio was 1, the result was reversed. The former was significantly lower than the latter at the same load mass and pressure angle. When the wheel-tooth ratio and the load mass were unchanged, the amplitude of each peak frequency of vibration acceleration decreased with the increase of pressure angle.

**Figure 15:**
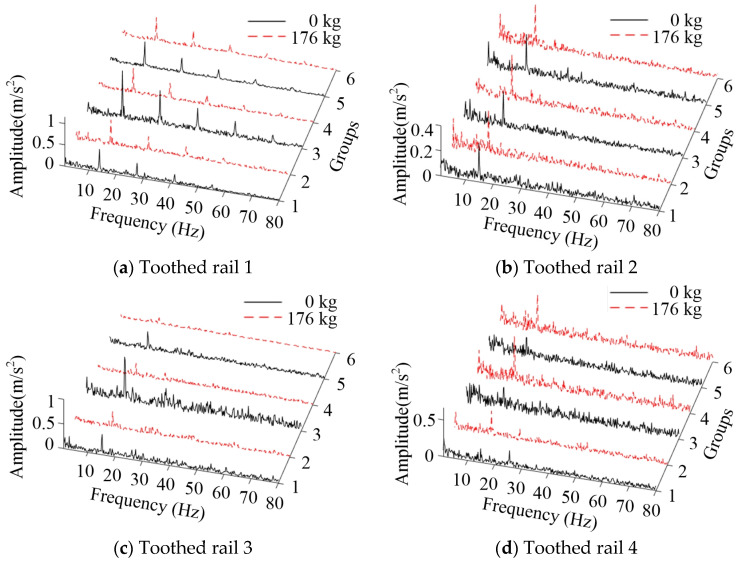
The Hilbert envelope demodulation frequency analysis of vibration acceleration.

The Hilbert envelope demodulation frequency analysis of vibration acceleration of each group is shown in Figure 15. Since there was no characteristic peak after 80 Hz, only 1~80 Hz was cut out for analysis. The same conclusions as above could be obtained.

### 4.2. Orthogonal Test Results and Analysis

The test results are shown in Table 5. Factor A is the pressure angle. Factor B is the wheel-tooth ratio. Factor C is the load mass. Factor A×B indicates an interaction between factor A and factor B, similarly for factor B×C and factor A×C. Factor A×B×C indicates an interaction between factor A, factor B, and factor C. The test results showed that the average maximum acceleration amplitude decreased with the decrease of the pressure angle. When the pressure angle was reduced from 25 degrees to 15 degrees, the average maximum acceleration amplitude was reduced from 5.5888 m/s^2^ to 4.4072 m/s^2^, which was reduced by 21.14%. The average attenuation time of the vibration was increased from 15.5788 ms to 16.5534 ms, an increase of 0.9746 ms. When the wheel-tooth ratio was changed from 1 to cosα, the average maximum acceleration amplitude was decreased from 5.6788 m/s^2^ to 4.3172 m/s^2^, with a decrease of 23.98%. However, the average attenuation time of vibration was increased by 1.8003 ms. When the load mass increased from 0 kg to 176 kg, the average maximum acceleration amplitude was decreased by 11.86%. Whereas, the average attenuation time of the vibration was increased by 0.1833 ms. The results showed that the maximum acceleration amplitude was decreased as a result of the pressure angle decreasing. As a consequence, the movement of the MSEMT was more continuous. When the wheel-tooth ratio was cosα, it moved much more smoothly than when it was 1. Furthermore, the average maximum acceleration amplitude decreased. The MSEMT also moved more smoothly with the increase of load mass. However, the average attenuation time of the vibration was not affected by the pressure angle, the wheel-tooth ratio, and the load mass. It was basically concentrated at 16.0662 ± 1.0019 ms.

**Figure 16:**
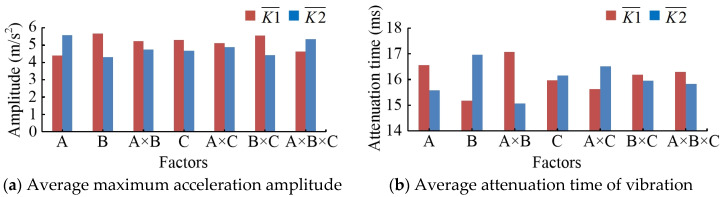
Range analysis results.

The range analysis results are shown in Figure 16a,b. K1 value is the sum of the results of various factors at level 1. So is K2. $\bar{K1}$ is the average value of K1, which is K1/4 in this paper. So is $\bar{K2}$. It was shown that the main factor impacting the average maximum acceleration amplitude was B, following by A, B×C, and A×B×C the last one, and the main factors of the average attenuation time of the vibration were sorted as A×B, B, A, and A×C. The main reason was that the wheel-tooth ratio and pressure angle had a direct impact on the variation of instantaneous velocity. This variation caused the impact of meshing. The results of the variance analysis are shown in Table 6. It was shown that the pressure angle and wheel-tooth ratio had a significant effect on the average maximum acceleration amplitude. So did the interaction between the wheel-tooth ratio and the load mass. Moreover, the pressure angle of 15 degrees was better than that of 25 degrees. The main reason was that the impact of kinetic energy was smaller with a pressure angle of 15 rather than 25. When the load mass remains constant, the wheel-tooth ratio of cosα was better than that of 1. It was because the variation of instantaneous velocity was smaller with the former situation than with the latter one. The wheel-tooth ratio and the interaction between the pressure and the wheel-tooth ratio had a highly significant effect on the average attenuation time of the vibration, as did the pressure angle and the interaction between the pressure angle and the load mass, but to a lesser extent. Hence, the optimal combination was B2A1C2, which means the wheel-tooth ratio was cosα, the pressure angle was 15 degrees, and the load mass was 176 kg.

## 5. Conclusions

The roller gear and rack mechanism, as the transmission mechanism of MSEMT, is an important part of the MSEMT. The rationality of it directly affects the mechanical performance, safety performance, and service life of MSEMT. The mechanism of meshing of the roller gear with involute toothed rail was studied in this paper. The displacement and instantaneous speed of MSEMT were modeled. It was concluded that the instantaneous velocity variation at the moment of meshing was dependent on the toothed rail parameters. However, the velocity variation led to the vibration of the meshing impact, which reduced the vibration smoothness of the MSEMT. The result of the simulation showed that the vibration frequency of the MSEMT occurred mainly in the meshing frequency and its octave, which suggested that the excitation source causing the vibration of the MSEMT was the impact of meshing.

Therefore, the meshing vibration test was carried out, and the vibration acceleration signals of the test were collected. To obtain the spectral band, the RCC method was applied. In addition, the bandpass filtered signals were analyzed by Hilbert envelope demodulation analysis. It was shown that there was a frequency doubling relationship between the main vibration frequency and the frequency of MRGTR, and the excitation source causing vibration of the MSEMT was MRGTR. The corresponding amplitude of the main frequency decreased with the increase of frequency doubling. Moreover, with the increase of pressure angle, the main vibration frequency was decreased and so was its amplitude. This results from the increase of the impulse, which is caused by the variation of velocity at the tooth-in process. The main vibration frequency as a wheel-ratio of cosα was less than that as a wheel-ratio of 1, and its amplitude was smaller as well. The orthogonal test results showed that the pressure angle and wheel-teeth of the toothed rail had a significant effect on the meshing vibration of the MSEMT, and the effect of the wheel-teeth ratio is greater than that of the pressure angle. Whereas, the load mass has no significant effect on the meshing vibration of the MSEMT. From the parameter selection of this experiment, it can be concluded that the meshing vibration acceleration is the smallest, and the vibration smoothness of the MSEMT is the best at the pressure angle of 15 degrees and the wheel-tooth ratio of cos*α*. The results of the simulation and test suggested that the parameters of the toothed rail had an effect on the vibration of the MSEMT. However, the result of the simulation had some deviations from the actual situation. The model and simulation parameters in this paper can be further improved to obtain more accurate simulation results.

The research provides a reference for improving the vibration smoothness of MSEMT and a theoretical basis for designing the toothed rail of MSEMT. However, there are still some shortcomings in this paper as well as no research on the influence of the machining accuracy and installation error of the toothed rail on the vibration of MSEMT, which leads to some deviations between the test results and the theoretical analysis. This will be the direction of our further research. Furthermore, the effects of vibration on other aspects, such as tracks, support structures, and slope foundations are worthy of research as well.

## Figures and Tables

**Table 1:** Rack parameters of each toothed rail.

Parameter	Toothed Rail 1	Toothed Rail 2	Toothed Rail 3	Toothed Rail 4
Pressure Angle *α* (°)	15	15	25	25
Pitch *p* (mm)	30.9	31.99	30.9	34.09
Wheel-tooth ratio	1	cos*α*	1	cos*α*
Height of tooth *hf* (mm)	35.5	35.5	35.5	35.5

**Table 2:** Parameters of the MSEMT.

Parameter	Numerical
Empty mass of MSEMT *M*_0_ (kg)	175
The rated speed of motor *n* (r/min)	1500
Transmission ratio *i*	17.143
Roller gear rotational speed *n_r_* (r/min)	81.67~93.33
Given speed *v_d_* (m/min)	25.65~29.32
Number of rollers *z*	10
Radius of the central circle of roller *R* (mm)	50
Radius of roller *r* (mm)	7

**Table 3:** Three factors and two levels of the test.

Levels	Factor A	Factor B	Factor C
	Pressure Angle *α* (°)	Wheel-Tooth Ratio	Load Mass *M*_2_ (kg)
1	15	1	0
2	25	cos*α*	176

**Table 4:** Peak amplitude-frequency table of the toothed rails.

Type	Load Mass (kg)	Peak Frequency (Hz)			Amplitude (m/s^2^)			Average Peak Frequency (Hz)	Average Amplitude (m/s^2^)
		Group 1	Group 2	Group 3	Group 1	Group 2	Group 3		
Toothed rail 1	0	13.87	13.87	13.87	0.5321	0.6117	1.1530	13.87	0.7656
		27.73	27.73	27.73	0.3490	0.3619	0.8353	27.73	0.5154
		41.6	41.6	41.6	0.2264	0.2679	0.5619	41.6	0.3521
		55.47	55.47	55.47	0.1258	0.1354	0.3972	55.47	0.2195
	176	13.67	13.67	13.67	0.5974	0.6102	0.5691	13.67	0.5922
		27.34	27.34	27.34	0.3838	0.3684	0.3977	27.34	0.3833
		41.02	41.02	41.02	0.2602	0.2355	0.2216	41.02	0.2391
		54.69	54.69	54.69	0.1459	0.1459	0.1398	54.69	0.1439
		68.36	68.36	68.36	0.1196	0.0966	0.1080	68.36	0.1081
Toothed rail 2	0	14.84	15.23	15.04	0.3093	0.3013	0.3325	15.04	0.3144
	176	14.06	14.06	14.06	0.3641	0.3762	0.3594	14.06	0.3666
Toothed rail 3	0	15.23	15.04	15.04	0.4167	0.9276	0.3825	15.10	0.5756
		27.54	30.27	30.27	0.2227	0.4285	0.1517	29.36	0.2676
	176	14.84	15.04	15.04	0.3438	0.2682	0.1348	14.65	0.2489
		29.78	25.78	28.91	0.1597	0.1652	0.0626	28.16	0.1291
Toothed rail 4	0	14.84	15.04	15.04	0.1911	0.3277	0.2997	14.97	0.2728
		25.59	28.52	32.03	0.2288	0.2224	0.1430	28.71	0.1981
	176	14.65	14.65	14.84	0.3534	0.6664	0.533	14.71	0.5176

**Table 5:** Orthogonal Test Results.

**No.**	**Factors**							
	**A** **Pressure angle**	**B** **Wheel-tooth ratio**	**A×B** **Interaction of pressure angle and wheel-tooth ratio**	**C** **Load mass**		**A×C** **Interaction of pressure angle and load mass**	**B×C** **Interaction of wheel-tooth ratio and load mass**	**A×B×C** **Interaction of pressure angle, wheel-tooth ratio and load mass**
1	1	1	1	1		1	1	1
2	1	1	1	2		2	2	2
3	1	2	2	1		1	2	2
4	1	2	2	2		2	1	1
5	2	1	2	1		2	1	2
6	2	1	2	2		1	2	1
7	2	2	1	1		2	2	1
8	2	2	1	2		1	1	2
**No.**	**Indicators**							
	**Average maximum acceleration amplitude (m/s^2^)**				**Average attenuation time of the vibration (ms)**			
1	5.9701				16.4678			
2	4.6906				16.8427			
3	3.7128				15.5683			
4	3.2553				17.3349			
5	7.1488				13.9068			
6	4.9058				13.4467			
7	4.4201				17.9550			
8	5.8806				17.0068			

**Table 6:** Analysis of variance results.

Analysis	Average Maximum Acceleration Amplitude (m/s^2^)				Average Attenuation Time of the Vibration (ms)			
	B	A	B×C	A×B×C	A×B	B	A	A×C
SS	3.708	2.792	2.560	1.038	8.031	6.482	1.900	1.575
df	1	1	1	1	1	1	1	1
MS	3.708	2.792	2.560	1.038	8.031	6.482	1.900	1.575
F	8.077	6.083	5.577	2.261	39.435	31.827	9.328	7.734
Sig.	0.066	0.090	0.099	0.230	0.008	0.011	0.055	0.069
significance	*	*	*		**	**	*	*

Note: SS = sum of squares; df = number of degrees of freedom; MS = mean square; F = the F test statistic; The critical value of F are 10.13 (*p* < 0.05) and 34.12 (*p* < 0.01); ** means significant at *p* < 0.01; * means significant at *p* < 0.05.
